# Oral Nanomedicines for siRNA Delivery to Treat Inflammatory Bowel Disease

**DOI:** 10.3390/pharmaceutics14091969

**Published:** 2022-09-19

**Authors:** Jongyoon Shinn, Juyeon Lee, Seon Ah Lee, Seon Ju Lee, Ah Hyun Choi, Jung Seo Kim, Su Jin Kim, Hyo Jin Kim, Cherin Lee, Yejin Kim, Joohyeon Kim, Jonghee Choi, Byungchae Jung, Taeho Kim, HyeonTaek Nam, Hyungjun Kim, Yonghyun Lee

**Affiliations:** 1 College of Pharmacy, Ewha Womans University, Seoul 03760, Korea; 2 Department of Chemistry and Bioscience, Kumoh National Institute of Technology, 61 Daehak-ro, Gumi 39177, Gyeongbuk, Korea

**Keywords:** oral drug delivery, siRNA, inflammatory bowel disease, gene delivery, nanomedicines, Immunotherapy

## Abstract

RNA interference (RNAi) therapies have significant potential for the treatment of inflammatory bowel diseases (IBD). Although administering small interfering RNA (siRNA) via an oral route is desirable, various hurdles including physicochemical, mucus, and cellular uptake barriers of the gastrointestinal tract (GIT) impede both the delivery of siRNA to the target site and the action of siRNA drugs at the target site. In this review, we first discuss various physicochemical and biological barriers in the GI tract. Furthermore, we present recent strategies and the progress of oral siRNA delivery strategies to treat IBD. Finally, we consider the challenges faced in the use of these strategies and future directions of oral siRNA delivery strategies.

## 1. Introduction

More than 3.5 million patients worldwide suffer from inflammatory bowel disease (IBD), which is characterized by chronic inflammation of the gastrointestinal (GI) tract [1,2]. IBD comprises two clinically distinct conditions: ulcerative colitis (UC), which is confined to the colon, and Crohn’s disease (CD), which affects any part of the GI tract, especially the ilium and the colon [3]. Even though UC and CD are considered different conditions, they share many clinical characteristics including persistent diarrhea, abdominal pain, and rectal bleeding/bloody stools. The exact cause of IBD remains unknown, but IBD-susceptible individuals typically have dysbiotic gut microbiota, disrupted intestinal barriers, and dysregulated mucosal immunity [4,5].

Aminosalicylates, corticosteroids, and immunosuppressive agents are common treatment options for IBD patients, although they produce side effects that can include depression, osteoporosis, and susceptibility to infection due to the non-specificity of drug action [6,7,8]. However, the non-specific action of such drugs has shown limitations such as non-target site toxicity and limited efficacy in the complete remission of the severe last stages of IBD [4]. Thus, targeted therapeutics, especially antibody therapeutics, directed to a more specific mechanism of action have led to reduced side effects as well as improved therapeutic efficacy [9].

IBD progression and development are highly associated with several genes and target cytokine dysregulations [10,11]. Some examples of dysregulated proteins involved in IBD are TNF-α, IFN-γ, IL-4, IL-10, and IL-21 [12,13]. For example, since most cells in the inflamed regions express receptors for TNF-α, it has promiscuous effects, rendering it an effective therapeutic target in IBD patients [14]. Thus, anti-TNF-α antibodies such as infliximab, adalimumab, certolizumab pegol, and golimumab have been widely used for the treatment of IBD [15], with enhanced therapeutic efficacy even in severe stages of IBD, while also limiting systemic toxicity [16,17,18,19]. However, antibody therapeutics present several limitations such as frequent painful injections and patient noncompliance. Furthermore, they have significant side effects including serious infections, malignancies, demyelinating disease, congestive heart failure, and the induction of autoimmune conditions due to systemic cytokine depletion [20,21,22,23].

As an alternative to systemic antibody therapeutics, small interfering RNAs (siRNAs) directed against proinflammatory cytokines have gained great attention from many research groups due to their potential to treat intestinal inflammation via the silencing of pro-inflammatory cytokines [24]. Various siRNA-based therapeutics are under development for the efficient treatment of inflammatory bowel diseases [25]. However, systemic treatment with siRNA-based therapeutics has many limitations such as patient non-compliance and systemic side effects caused by the systemic depletion of cytokines [26], which has encouraged the development of strategies for the specific delivery of siRNA to the desired inflamed site of the GI tract.

Compared with other routes, oral administration is one of the most commonly used drug administration routes because of high patient compliance, simple self-administration, non-invasiveness, and low cost [4,27,28,29]. For macromolecular drugs, including siRNAs, most are prone to degradation and the loss of bioactivity from the harsh GI tract conditions such as pH variation and digestive enzymes. Thus, few orally administered macromolecular IBD drugs reach the target site of the colon [4,30,31,32]. Furthermore, penetrating lipid membranes of a target cell are another challenge for macromolecular drugs, including siRNA directed to intracellular target molecules (e.g., cytosolic mRNA for siRNA). Therefore, there is a great need for oral drug delivery systems with a demonstrated ability to protect siRNA from the harsh environment of the GI tract and selectively deliver siRNA to desired target sites—both the inflamed site and the intracellular cytosolic region [33].

In this review, we first discuss various physicochemical and biological barriers in the GI tract and strategies to overcome them. Furthermore, we present the recent progress of oral siRNA delivery strategies to treat IBD. Finally, we consider challenges facing the use of these strategies and future directions for oral siRNA delivery strategies.

## 2. Physiological and Biological Barriers and Strategies for the Oral Delivery of siRNA Drugs

The GI tract can be divided into two main parts: upper and lower sections. The upper GI tract consists of the oral cavity, pharynx, esophagus, stomach, and the first section of the small intestine (duodenum). The lower GI tract includes the rest of the small intestine (jejunum and ileum) and the large intestine segments (cecum, colon, and rectum) [34]. The lumen of the GI tract is lined by smooth muscle cells, covered by mucus, submucosa, and several muscle layers of epithelial cells, lamina propria, and muscularis mucosae [35]. Drug absorption mostly occurs in the small intestine due to a large absorption area and long residence time. The environmental conditions that influence drug stability and absorption include pH, enzymes, mucus barriers, cellular permeability barriers, the residence time of the drug, and GI surface area [36,37,38]. In addition, IBD can induce changes in pH, the mucus barrier, and the permeability of the intestinal epithelium in the GI tract, barriers that should be considered in the development of efficient drug delivery systems [39,40,41].

Large differences between the stomach, small intestine, and large intestine in terms of pH (Stomach: pH 2–4, Small Intestine: pH 6–7.4, Large intestine: pH 6 (Normal), pH 2.3–5.5 (IBD)), transit time (Stomach: 2–4 h, Small Intestine: 2–6 h, Large Intestine: 20–51 h (Normal) 10–24 h (IBD)), and gut microbiota conditions (duodenum: 10^3^–10^4^; jejunum: 10^4^–10^5^; ileum: 10; large intestine: 10^11^) have been utilized for conventional drug delivery formulations such as Pulsincap and CODES, which release loaded drugs in a pH-, transit time-, and/or gut microbiota-dependent manner [4,42]. However, conventional oral drug delivery formulations have limited efficacy and specificity for diseased colon tissue versus healthy colon tissue [43]. Despite the coverage of the colonic surface (including diseased tissue), there is no guarantee that a drug will be effectively taken up by the tissue and cells at the site of inflammation [42]. Additionally, even though inflammation can develop in the small intestine in Crohn’s disease patients, conventional oral drug delivery formulations usually target the colon but not the small intestine [4,42]. Furthermore, none of the conventional formulations have yielded a good response for the targeted and intracellular delivery of siRNA drugs due to the lack of inflamed cell targeting and intracellular deliverability. Therefore, there is a great need for oral drug delivery systems that demonstrate the ability to protect siRNA from the harsh environment of the GI tract and selectively deliver siRNA to desired target sites—both the inflamed site and the intracellular cytosolic region.

**Figure 1:**
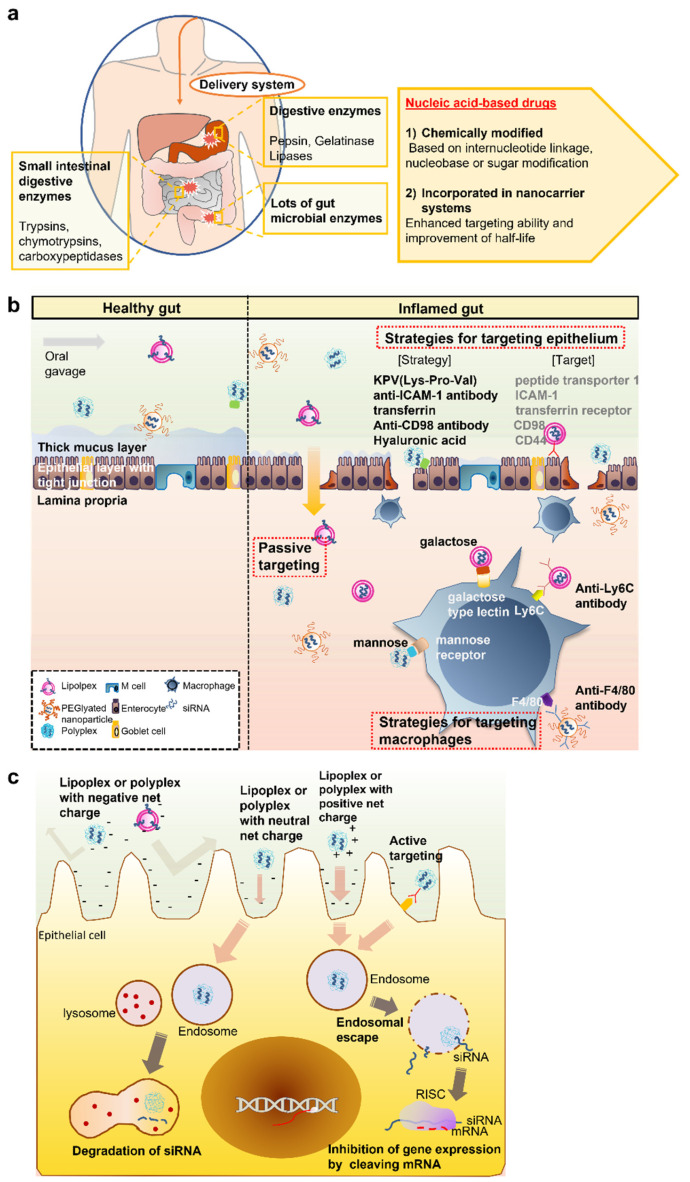
Physiological and biological barriers and strategies to orally deliver siRNA drugs. (**a**): Biochemical barriers and strategies to overcome them. siRNA can be degraded by various enzymes in the GI tract. Through chemical modification or encapsulation in nanocarriers, siRNA can be protected from degradation. (**b**): Intestinal barriers and strategies to overcome them. Tight junctions between epithelial cells and the thick, negatively charged mucus layer hinder the penetration of nanoparticles. However, in IBD patients, because of the significantly reduced mucus amount, the penetration of drug-loaded nanoparticles is much easier than through the mucus layer of the healthy gut. Forming a polyplex or lipoplex composed of cationic lipids with partial negative/neutral charges could be an effective way to overcome this limitation. Further functionalization for targeting ligands with intestinal epithelium and macrophages present on the nanoparticles could improve targeting to the site. (**c**): Cellular uptake barriers and strategies to overcome them. A high density of negative charges on the target cells prevents nanoparticles from penetrating cellular membranes. Even though nanoparticles are taken up by cells, siRNA can be degraded by lysosomal enzymes unless nanoparticles escape from the endosomes. A positive net charge of the polyplex or lipoplex can induce effective intracellular delivery with efficient endosomal escape activity. Nanoparticles with targeting ligands can be taken up by specific cells regardless of their charge.

Harsh gastrointestinal conditions, intestinal barrier (both mucus and inflamed epithelium) penetration issues, cell-targeting ability, and intracellular penetration are significant issues for the successful inflamed tissue/cell-specific delivery of siRNA-loaded drug delivery systems compared to conventional oral drug delivery formulations that usually exploit pH variances, transit time differences, and gut microbiome variations between the stomach, small intestine, and large intestine [44]. The details of each obstacle and strategies to overcome them will be discussed in the following sections (Table 1 and Figure 1).

### 2.1. Biochemical Barriers

For the successful treatment of disease by oral delivery, the system needs to overcome major biochemical barriers such as enzymatic degradation (Table 1 and Figure 1a). In the stomach environment, digestive enzymes such as pepsin and gelatinase can degrade biological therapeutics. Lipases in the stomach can also lead to the hydrolysis of drugs with hydrophobic lipid-related portions. The small intestinal environment is another biochemical barrier that needs to be considered. The high levels of small intestinal digestive enzymes such as trypsins, chymotrypsins, carboxypeptidases, and elastases are often responsible for the degradation and loss of bioactivity of macromolecular drugs such as siRNA drugs [45]. In contrast, the colon offers a longer residence time, relatively neutral pH values (6–6.7), and low concentrations of digestive enzymes [46]. However, there are many gut microbial enzymes that pose additional risks to the successful delivery of siRNAs.

To avoid degradation by nucleases, orally administered nucleic acid-based drugs have been either chemically modified to improve stability against enzymes or incorporated into nanocarrier systems to shield against enzymatic attacks (Table 1 and Figure 1a). The chemical modification of siRNA drugs is typically based on internucleotide linkage, nucleobase, or sugar modification. For example, endo-and exo-nucleases target internucleotide phosphodiester linkages of DNA and RNA. Thus, internucleotide linkage modifications such as N3′ phosphoramidates, phosphorothioates, boranophosphate 2′,5′–phosphodiesters, morpholinos, peptide nucleic acids, and amide-linked phosphonoacetates are mainly used to increase the resistance of siRNA to enzymatic attacks [47]. Chemical modification is generally sufficient to protect small-sized siRNA drugs against systemic metabolism, leading to the enhancement of drug activity [47]. On the other hand, the co-administration of nuclease inhibitors such as Ethylenediamine tetraacetic acid (EDTA)**,** sodium dodecyl sulfate, and aurintricarboxylic acid with siRNA drugs is another strategy to improve stability [48]. To localize the inhibitory activity, these inhibitors could be immobilized onto polymers as one of the components of the drug carrier matrix. The chitosan-EDTA conjugate is an example of such a system [48].

In most cases, siRNA drugs are rapidly excreted due to a lack of inherent targeting ability and small size. Thus, other strategies including nanomedicine-based protection have gained much attention from siRNA researchers because of various advantages, including enhanced targeting ability and the improvement of half-life [26] (Table 1 and Figure 1a). Another advantage of nanoparticle-based strategies is the enhanced intracellular delivery of siRNA, although this is not guaranteed by the above strategies. In particular, siRNA drugs that are not sufficiently stabilized by chemical modifications and larger siRNA drugs should apply nanomedicine-based protection strategies. The nano-complexation (polyplex or lipoplex) of siRNA drugs with various artificial cationic materials such as polyethyleneimine (PEI), 1,2-dioleoyl-sn-glycero-3-phosphate (DOPA), 1,4,7,10-tetraazacyclododecane1,4,7,10-tetraacetic acid (DOTA), or 1,2-dioleoyl-3-trimethylammonium-propane(DOTAP) or natural polymers such as chitosan, thiolated chitosans, trimethyl chitosan, or glycol chitosan leads to the obstruction of the free access of nucleases to nucleic acid-based therapeutics, probably due to increased steric hindrance via nanoparticle formation [44]. Utilizing cationic lipid liposomes is another strategy to improve the resistance of siRNA degradation by nucleases [49]. Another potential strategy is self-emulsifying drug delivery systems (SEDDS) [50]. The incorporation in SEDDS composed of oil droplets after the hydrophobic ion pairing of siRNA drugs with cationic lipids led to protection against nuclease attacks due to the limited penetration of nucleases into the oily droplets. Nanoparticle-embedment in hydrogels and other additional materials could further impede the free access of nucleases to siRNA drugs of a polyplex or lipoplex, leading to further protection against nuclease attacks.

### 2.2. Intestinal Barrier: Mucus and the Intestinal Epithelium

The intestinal barrier is composed of a single layer of intestinal epithelial cells and a mucus layer. In healthy conditions, mucus is a highly hydrated gel comprised of 95% water, 5% mucin glycoprotein, and minor components such as lipids, mineral salts, and proteins that contribute to mucus elasticity [51,52]. The mucus layer has many components stabilized by covalent and noncovalent interactions, including hydrogen bonds and electrostatic, hydrophobic, or other interactions, creating a mesh network that affects the penetration of molecules and drugs and their diffusion rates [53,54]. Mucin fibers are polymeric gel-forming glycoproteins of a high molecular weight (10–40 MDa) secreted by goblet cells and submucosal glands of the lamina propria at the apical epithelium [55]. Mucins are filamentous O-linked glycoproteins with repeated domains of amino acids such as proline, threonine, and serine, which are highly glycosylated, with a carbohydrate density greater than 70% [56,57]. Due to their carbohydrate density, mucins are crosslinked in a brush-like structure [53]. At acidic pH, mucins undergo a conformational change from a random coil to extended conformation and form a gel phase in mucus, resulting in a sol-gel transition state [58]. In addition, ionic strength changes may initiate the formation of a gel phase in mucus, which can promote the assembly of mucins into large linear or branched aggregates [59]. Therefore, physicochemical characteristics such as pH, ionic strength, composition, and conformation are important in the formation and function of mucus.

Nanomedicines have to be smaller than 100 nm and muco-inert to penetrate the mucus gel layer. Forming a polyplex composed of nucleic acid-based therapeutics and cationic polymers or a lipoplex composed of siRNA drugs and cationic lipids with partial negative/neutral charges could be an effective way to overcome this limitation. Typically, nanoparticles are formed with an average size less than 100 nm and are comparatively slippery in the mucus layer due to a high density of charges on the surface [60]. Even though a Polyethylene glycol (PEG) coating might hinder the intracellular uptake of nanomedicines, it is another strategy to promote mucus penetration due to its anti-biofouling effects [61,62,63]. Other strategies utilize mucolytic agents such as sulfhydryl compounds (e.g., dithiothreitol, N-acetyl-cysteine) [64]. In fact, in IBD patients, significant changes in mucus composition, thickness, physical properties, and function occur. IBD patients have significantly reduced mucus, resulting in the decreased thickness of the mucus layer [4]. In many cases, the mucus layer penetration of drug-loaded nanoparticles is much easier than that of the mucus layer of a healthy gut. Thus, strategies based on lipoplexes, polyplexes, or PEGylation are common for the specific penetration of the thin or denuded mucus of the inflamed gut. However, utilizing mucolytic agents is not a good option because the non-specific removal of the mucus layer affects the non-specific accumulation of nanoparticles into healthy tissue, and the non-restricted passage of pathogens and other molecules could occur (Table 1 and Figure 1b).

If target cells such as immune cells are located underneath the intestinal epithelium, the nanoparticles must also penetrate the intestinal epithelium [4]. The intestinal epithelium is the outermost layer of cells composed of tight junctions and three kinds of cells: enterocytes, goblet cells, and microfold cells (M-cells) [65]. Enterocytes, or intestinal absorptive cells, are the most abundant cells of the epithelium layer and line the inner surface of the intestines, enhancing the transportation of nutrients and water from lumen to the bloodstream. Goblet cells that comprise 10–20% of epithelial cells, ranging from 4% in the duodenum to 16% in the distal colon, produce a thick two-layered mucous intestinal lining [35,66,67,68]. M-cells are specialized intestinal epithelial cells that cover the Peyer’s patches and are capable of taking up antigens from the lumen and transporting them intact into the underlying lamina propria [69]. Tight junctions between intestinal epithelial cells are paracellular barriers that play a crucial role in gut homeostasis and intestinal barrier function [70].

In addition to the denuded mucus layer, IBD patients also have a disrupted intestinal epithelium nearby inflamed sites [4,71]. When the integrity of cell–cell junctions is disrupted by diverse pathological states, autoimmune diseases such as IBD and systemic infection with the unrestricted passage of pathogens and molecules across the epithelial layers can occur [4,71]. This phenomenon usually affects unregulated mucosal immune responses against gut microbiota. Thus, orally administered nano-based drug carriers could accumulate in the inflamed sites via both the denuded mucus layer and the disrupted intestinal epithelium (Table 1 and Figure 1b). In contrast, the carriers are rarely localized in the healthy gut due to the intact mucus layer and tight intestinal epithelium [72]. This leads to the specific delivery of oral nanomedicines to inflamed tissue [72]. The further functionalization of targeting ligands on the nanoparticles could improve drug targeting to the sites [72] (Table 1 and Figure 1b). Targeting intestinal epithelium and macrophages is a common active targeting strategy. For epithelium targeting, KPV (Lys-Pro-Val; for targeting peptide transporter 1) [73,74], anti-intercellular adhesion molecule-1 (ICAM-1) antibody (for targeting ICAM-1) [75], transferrin (for targeting transferrin receptor) [76], anti-CD98 antibody (for targeting CD98) [77], and hyaluronic acid (for targeting CD44) [5,78] have been utilized. For immune cell (e.g., macrophage) targeting, mannose (for targeting mannose receptors) [79], galactose (galactose-type lectin) [80], anti-F4/80 antibody (for targeting F4/80) [81], and anti-Lymphocyte antigen 6 complex (Ly6C) antibody (for targeting Ly6C) [82] have been explored [4]. After targeting, nanoparticles must be taken up by target cells such as epithelial cells and immune cells to allow for siRNA activity, which will be discussed in the next section.

### 2.3. Cellular Uptake Barrier

When siRNA drugs or nanoparticles carrying siRNA drugs reach target cells such as epithelium and immune cells, siRNA drugs or nanomedicines must penetrate cellular membranes to silence target molecules (e.g., mRNA) in the cytosol [26,83]. For effective siRNA drug penetration through cellular membranes, the most challenging barrier is a high density of negative charges on the target cells such as intestinal epithelium cells and immune cells [26,83]. The cellular membrane is typically composed of negatively charged phospholipids in a bilayer, which disrupt siRNA intracellular delivery due to repulsive interactions between the negative charges of both siRNA and cellular membranes [26,83] (Figure 1c and Table 1).

**Figure 2:**
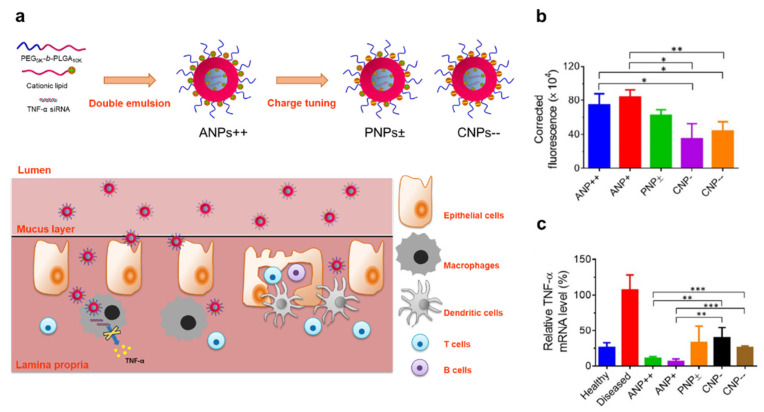
Oral siRNA nanotherapeutics based on polyplexes with a positive net charge show better targeting/therapeutic efficacy than those with a neutral/negative net charge. (**a**): Illustration of a methodology to modify the surface charge of siRNA-encapsulating polymeric NPs, highlighting the influence of surface charge on the oral delivery of siRNA for localized inflammatory disorders. (**b**): Aminated nanoparticles (ANPs) show a better targeting ability than plain NPs (PNPs) and carboxylated NPs (CNPs). (**c**): ANPs exhibited better therapeutic efficacy in a DSS-induced murine colitis model than PNPs and CNPs. Statistical *p*-values: * p < 0.05, ** *p*  < 0.01, *** *p *< 0.001. Reprinted/adapted with permission from Ref. [89]. 2018, Springer Nature.

Lipoplexes or polyplexes formed between nucleic acid-based therapeutics and cationic lipids or polymers could fuse with cellular membranes due to increased lipophilicity, leading to enhanced intracellular uptake. The final fate of the lipoplex or polyplex depends on the net charge of the complex (Table 1 and Figure 1c). A strong negative net charge of the lipoplex or polyplex could hinder the intracellular uptake of the nanoparticles from charge–charge repulsion, although lipophilicity is increased [44]. Even though a neutral net charge of the lipoplex or polyplex is more effective in mucus penetration and cell membrane penetration because of increased lipophilicity, the escape from endo/lysosomal vesicles is the limiting step for these entities [26,83,84]. Since siRNA drugs are very vulnerable to lysosomal enzymes, nanoparticles carrying siRNAs need to escape from the endosomes before they fuse with lysosomes or are recycled back to the membrane [26,61,83]. A positive net charge of the polyplex or lipoplex with or without an additional endosomal escape moiety could induce effective intracellular delivery together with efficient endosomal escape activity [83,85,86]. However, in many cases, the complex can be trapped in the mucus layer [87,88]. However, considering mucus layers are thin or denuded in the inflamed region, higher levels of cationic nanoparticles could be localized in the inflamed site underneath the mucus layer compared to healthy sites [4,89]. Therefore, most currently developed oral nanomedicines carrying siRNA drugs are based on lipoplexes or polyplexes that form a positive net charge between nucleic acid-based therapeutics and cationic lipids or polymers. These seem to be more promising than the neutral poly/lipoplex or negative poly lipoplex. In a study supporting this demonstration, Iqbal et al. prepared poly/lipoplexes composed of PEG- poly(lactic-co-glycolic acid) poly(lactic-co-glycolic acid) (PLGA), cationic lipid, and TNF-α siRNA, with three kinds of charge statuses (cationic poly/lipoplex, neutral poly/lipoplex, and positive charge poly/lipoplex) [89] (Figure 2a). Notably, the cationic poly/lipoplex showed better inflamed colon-targeting ability with potent anti-inflammatory activity in the dextran sodium sulfate (DSS)-induced colitis model compared to the neutral poly/lipoplex and negative poly/lipoplex (Figure 2b,c).

When reaching target sites, nanoparticles without the functionalization of targeting ligands could be non-specifically internalized into many kinds of cells after the penetration of the mucus layers. In comparison, active targeting functionalized nanoparticles are taken up by specific cell types such as inflamed epithelial and activated macrophages (please see the previous part for the targeting ligand counterparts) [4]. In these cases, a cationic net charge is not necessary because intracellular uptake can occur via receptor-mediated endocytosis (Table 1).

Cationic nanoparticles based on polyplexes and lipoplexes could be non-specifically bound to healthy intestinal barriers due to charge–charge interactions, as mentioned above. To minimize non-specific binding to the healthy intestinal barriers, to enhance protection against harsh gastrointestinal environments, and to improve the penetration of the leaky inflamed intestinal barrier, hydrogel or additional polymeric nanoparticle encapsulation strategies could be utilized. In these strategies, the polyplex or lipoplex should be liberated from the embedding materials via the controlled release of the loaded polyplex or lipoplex. Stimuli (pH/enzyme/reactive oxygen species (ROS))-responsive polymer-based nanoparticle-encapsulation methods could be utilized for this strategy.

## 3. Oral siRNA Delivery for IBD Treatments

To date, various gastrointestinal tract (GIT) barriers for the inflamed site-specific delivery of siRNA drugs and strategies to overcome limitations with oral nanomedicine encapsulation have been exploited. Orally administered nanomedicines should overcome the barriers with the various strategies discussed in previous sections. In this section, examples of oral nanomedicines carrying siRNA drugs will be discussed by category: (1) Polyplex or lipoplex. (2) Polyplex or lipoplex embedded in hydrogels or polymeric materials (Table 2).

### 3.1. Polyplex or Lipoplex

**Figure 3:**
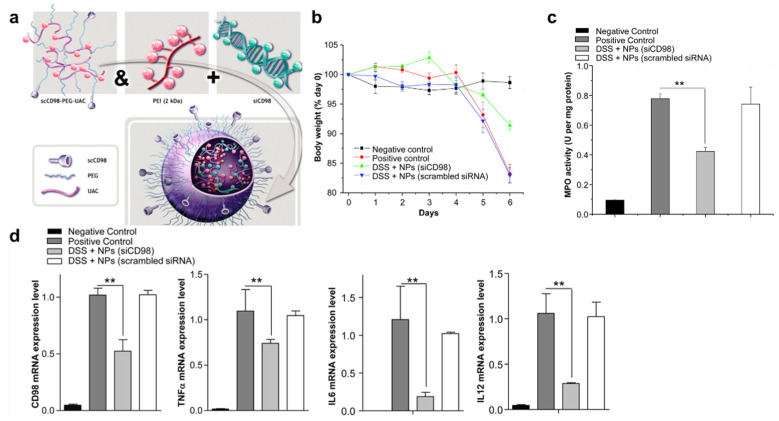
Oral CD98 siRNA-loaded nanotherapeutics decrease the severity of colitis in mice. (**a**): Schematic representation of the self-assembly of single-chain CD98 antibody-functionalized siRNA-loaded nanoparticles. (**b**): Body weight in DSS-induced colitis mice. (**c**): Colonic Myeloperoxidase (MPO) activity. (**d**): The mRNA levels of CD98 and proinflammatory cytokines in mice by treatment. Statistical *p*-values: ** *p* < 0.01. Reproduced with permission [77]. 2014, Elsevier.

The nano-complexation of siRNA drugs with cationic lipids (lipoplex) or polymers (polyplex) endows the protection of siRNA against the harsh conditions of the GIT. Furthermore, the drug could be localized in the inflamed site via denuded mucus and leaky intestinal epithelium. The polyplex or lipoplex can easily penetrate into the remaining mucus layer of the inflamed tissue due to the comparatively smooth movement caused by a high density of charges at the surface. At the inflamed target site, the polyplex or lipoplex can be non-specifically taken up by intestinal epithelial cells or diverse immune cells (sometimes, even anti-inflammatory immune cells). For example, a polyplex—polymeric micelles (150–300 nm) composed of amphiphilic poly-allylamine complexed with an siRNA drug—was developed [90]. The polyplex was very stable in simulated gastric/intestinal fluids. In addition, the polyplex exerted intracellular uptake followed by efficient endosomal escape while showing potent gene knockdown activity in Caco-2 cells. As an example of a lipoplex, the commercially available transfection agent, lipofectamine, is a representative positively charged lipid that facilitates binding and fusion with the cellular membrane to release the complexed siRNA into cells [91,92]. Even though the transfection activity is very effective, toxicity is a limitation for the general application of lipofectamine [93]. To overcome the limitation of synthetic lipids such as lipofectamine, biocompatible non-toxic natural source-derived cationic lipoplexes could be utilized. Zhang et al. developed ginger-derived lipid nanoparticles (GDLNs) complexed with CD98 siRNA [94] for the treatment of a DSS-induced murine colitis model. GDLNs complexed with CD98 siRNA exhibited effective gene silencing under in vitro conditions (Colon-26 cells (inflamed epithelium cells) and RAW 264.7 cells (macrophage cells)) and in vivo conditions (the ileum and colon). Notably, orally administered GDLNs showed better therapeutic efficacy than the systemic administration of naked CD98 siRNA [77] (Figure 3).

As demonstrated earlier, PEGylation can enhance mucus penetration and siRNA protection due to its anti-biofouling action. PEGylated lipoid nanoparticles (LNPs) composed of cholesterol, Distearoylphosphatidylcholine (DSPC), and PEG-lipid were developed to target inflamed intestinal epithelial cells while showing gene silencing [94,95]. The PEGylated LNPs complexed with Glyceraldehyde-3-Phosphate Dehydrogenase (GAPDH) siRNA were stable in harsh environments, such as pH variation and enzymatic degradation, and showed the ability to target intestinal epithelial cells. However, GAPDH silencing in vivo was not statistically significant compared to the control group, indicating that PEGylation might inhibit the intracellular uptake of the nanoparticles. Thus, for this strategy, active targeting functionalization could be required to improve intracellular delivery. On the other hand, in other studies, PEGylated poly/lipoplexes carrying TNF-α siRNA showed the opposite activity. Combining a cationic lipid and PEG-PLGA polymer with TNF-α siRNA led to cationic PEGylated poly/lipoplexes [89]. PEGylated poly/lipoplex TNF-α carrying siRNA were very stable in gastric and intestinal fluid and RNase A. Of note, the cationic poly/lipoplex showed good inflamed colon targeting with potent anti-inflammatory activity in the DSS-induced colitis model.

**Figure 4:**
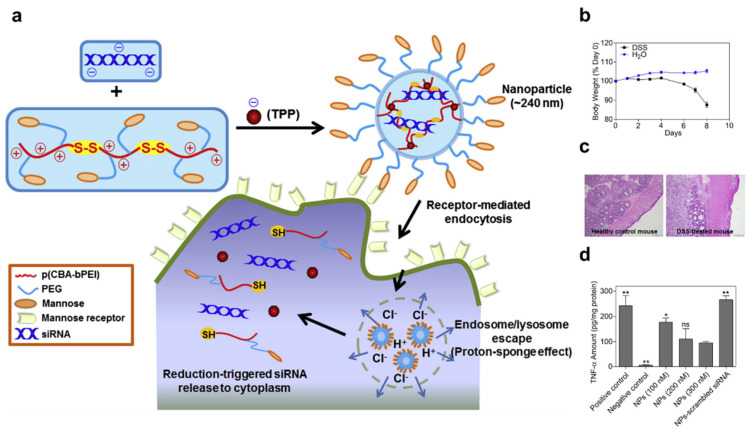
Active-targeting functionalized polyplex-based nanomaterials. (**a**): Schematic illustration of TPP-PPM/siRNA nanoparticle (NP) formation, macrophage-targeting delivery, and release of siRNAs to the cytoplasm (**b**): Changes in body weight over time in mice treated with 3% DSS in drinking water. (**c**): Hematoxylin− and eosin-stained colon sections from healthy control and DSS-treated mice sacrificed on day 8. (**d**): Inhibition of TNF-α secretion by siTNF-loaded triphosphate-cationic polymer nanoparticles (TPP-PPM/siTNF NPs) (weight ratio, 40:1) at different siTNF concentrations (100 nm, 200 nm, and 300 nm). Statistical *p*-values: No significance: ns; * *p *< 0.05, ** p < 0.01. Reproduced with permission [98]. 2013, Elsevier.

The functionalization of active targeting ligands on the nanomaterial could induce target cell-specific distribution and enhance cellular membrane penetration via receptor-mediated endocytosis. Targeting receptors (mannose receptor [96] and galactose-type lectin [97]) on macrophages, known to be involved in gut inflammation, have also been investigated as a means of enhancing the oral delivery of siRNA. Mannosylated nanoparticles formulated using a cationic polymer and containing TNF-α siRNA were effectively taken up in vitro by macrophages and inhibited protein expression in colitis tissue ex vivo [98] (Figure 4). The oral administration of galactosylated trimethyl chitosan-cysteine nanoparticles loaded with Map4k4 siRNA decreased the severity of inflammation in a DDS colitis mouse model [80]. In these cases, net-negative charge nanoparticles could also be delivered into the intracellular region.

Even though these strategies have shown good efficacy in both in vitro and in vivo studies, there is a possibility for the lipoplex or polyplex to non-specifically bind to healthy intestinal barriers, especially the mucus layer. As the layer is typically composed of negatively charged molecules, complexes may not easily penetrate into the healthy mucus layer [99]. Furthermore, non-specific binding/trapping of the lipoplex or polyplex to/in the healthy tissue layer may compromise the therapeutic efficacy of the nanoparticles.

### 3.2. Polyplex or Lipoplex Embedded in Hydrogels or Polymeric Materials

**Figure 5:**
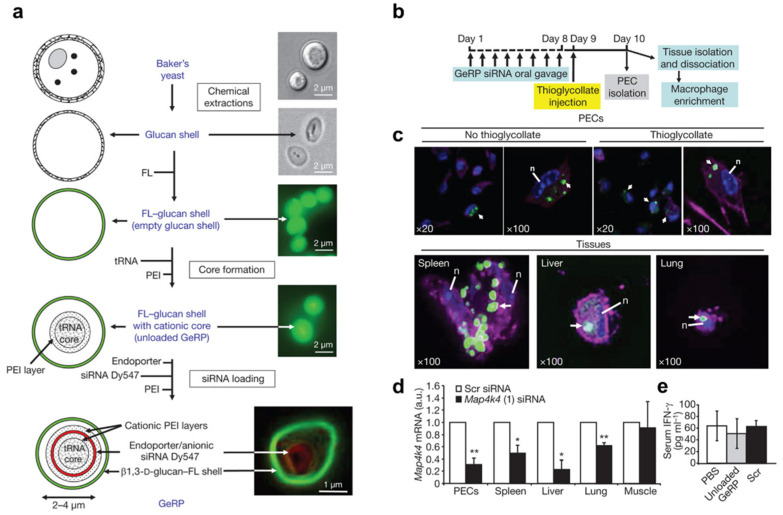
Polyplex embedded in polymeric materials. (**a**): Production of fluorescent GeRPs. (**b**): Timeline of GeRP (scrambled (Scr) or Map4k4, oligo (1)) administration and PEC/tissue isolation. (**c**): Confocal microscopy of PECs and tissue macrophages containing GeRPs (green). (**d**): Map4k4 mRNA expression in polyelectrolyte complexes (PECs) and adherent cells from tissues. Statistical *p*-values: * p < 0.05, ** p < 0.01. (**e**): Serum INF-γ levels from mice gavaged with PBS, unloaded GeRPs, or GeRPs containing 20 µg·kg^−1^ of Scr siRNA. Reproduced with permission [103]. 2009, Springer Nature.

To minimize the non-specific binding of the cationic lipoplex or polyplex, embedment into additional materials such as polymeric nanoparticles, microparticles, lipid droplets, or hydrogels could be utilized. These additional materials also endow the formulation with improved stability by providing an additional protective layer. Even though the embedment of the polyplex or lipoplex into stable materials increases the stability of loaded siRNA drugs in the harsh environment and non-specific binding of the polyplex or lipoplex into healthy tissue, robust loading of the polyplex or lipoplex could hamper the efficient release of loaded materials from the embedding materials. Thus, stimuli (pH, enzymes)-responsive polymers have been generally utilized for the target site-specific delivery of the loaded polyplex or lipoplex. For example, poly(epsilon-caprolactone) (PCL) microspheres were used to entrap type B gelatin nanoparticles encapsulating TNF-α siRNA (nanoparticles-in-microsphere oral system(NiMOS) formulation) [100]. The NiMOS formulation remained stable under acidic gastric conditions and released the gelatin nanoparticles only in the intestinal pH in the presence of lipase. Loaded siRNA was also very stable against RNase due to the dual nano/microstructures. The formulation also showed promising gene silencing while exerting anti-inflammatory activity in the DSS-induced murine colitis model. This formulation was also effective for the oral delivery of pDNA(plasmid DNA) [101]. A TNF-α siRNA-loaded pH-responsive nanogel encapsulated in a trypsin-mediated degradable microgel (P[Methacrylic acid (MAA)-co-N-Vinylpyrrolidone (NVP)] cross-linked with a trypsin-degradable peptide linker) protected the nanogel from release in the gastric acidic pH, while the nanogel was released by trypsin in the intestine. The released cationic nanogels could penetrate the mucus layer through the PEG corona and be taken up by macrophages, followed by endosomal escape via the cationic Diethylaminoethyl Methacrylate (DEAEMA) polymer, resulting in the knockdown of TNF-α [102]. The β1,3-D-glucan-shell encapsulating polyplex composed of Map4k4 siRNA complexed with polyethylenimine (PEI) (Glucan-Encapsulated siRNA Particles (GeRPs)) is very stable in stomach and intestinal conditions, as well as against nuclease attacks [103]. GeRPs also show a high uptake by phagocytic cells and low non-specific binding en route to the gut. Thereby, GeRPs exhibited potent gene silencing activity and significant protective activity against lipopolysaccharide (LPS)-induced colitis in a mouse model (Figure 5).

**Table 2:** Oral siRNA nano-delivery systems for IBD treatment.

siRNA Delivery Methods		Nanomaterials	siRNA	Target	References
Lipo/polyplex formation	-	PEGylated poly/lipoplex	TNF-α siRNA	DSS-induced colitis (in vivo)	[89]
		Polymeric micelles	Luciferase GL3 siRNA	Caco 2 cell (in vitro)	[90]
		Ginger-derived lipid nanoparticle	CD98 siRNA	Colon-26 and RAW 264.7 cells (in vitro), CD98 mRNA expression in intestinal tract (in vivo)	[94]
	With targeting ligands	Galactosylated trimethyl chitosan-cysteine nanoparticle	Map4k4 siRNA	Raw 264.7 cell (in vitro), DSS-induced colitis (in vivo)	[80]
		Mannosylated nanoparticle	TNF-α siRNA	Raw 264.7 cell (in vitro), DSS-induced colitis (ex vivo)	[98]
Lipo/polyplex embedded in hydrogels or polymeric materials	-	Type B gelatin nanoparticle in PCL microsphere	TNF-α siRNA	DSS-induced colitis (in vivo)	[100]
		pH-responsive nanogel in trypsin-mediated degradable microgel	TNF-α siRNA	Raw 264.7 cell (in vitro)	[102]
		1,3-D-glucan-shell polyplex in PEI	Map4k4 siRNA	LPS-induced colitis (in vivo)	[103]
	With targeting ligands	PEI polyplex in chitosan/alginate hydrogel with scCD98 antibody	CD98 siRNA	Colon-26 and RAW 264.7 cells (in vitro), DSS-induced colitis (in vivo)	[77]
		PEG-PLA nanoparticle in chitosan/alginate hydrogel with anti-F4/80 antibody	TNF-α siRNA	Raw 264.7 cell (in vitro), DSS-induced colitis (in vivo)	[81]

**Figure 6:**
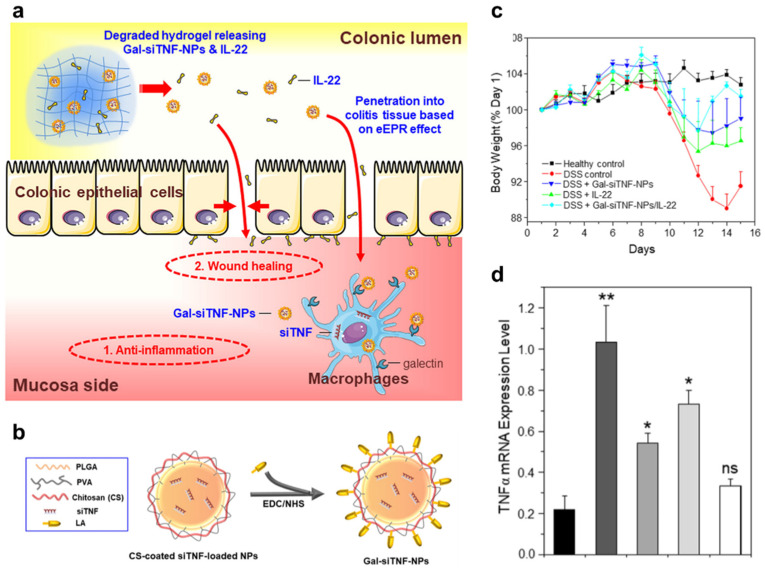
Polymer-based nanoparticles embedded in hydrogels. (**a**): Oral administration of Gal-siTNF-nanoparticles (NP)s plus IL-22 embedded in a hydrogel (chitosan/alginate) shows a much stronger capacity to down-regulate the expression of pro-inflammatory factors and promote mucosal healing. (**b**): Schematic illustration of the preparation of Gal-siTNF-NPs. (**c**): Mouse body weight over time, normalized as a percentage of day-one body weight. (**d**): TNF-α mRNA expression levels in different mouse treatment groups. Statistical *p*-values: No significance: ns; * p < 0.05, ** p < 0.01. Reproduced with permission [104]. 2018, Elsevier.

Functionalization with active targeting ligands on the loaded nanomaterials could also improve target cell-specific distribution after the release of the nanomaterials from the embedding material. In addition, enhanced cellular membrane permeation could also be expected via receptor-mediated endocytosis. In these cases, a positive net charge is not necessary. For example, anti-F4/80 antibody-functionalized PEG-PLA nanoparticles carrying TNF-α siRNA complexed with polyethyleneimine were encapsulated in a chitosan/alginate hydrogel to improve the stability and siRNA delivery of the nanoparticles [81]. The hydrogel collapses in intestinal solutions at pH 5 or 6, which reflects the colonic pH under inflamed and non-inflamed states, leading to the specific release of loaded nanoparticles in the colonic lumen. Finally, the released nanoparticles (almost neutral net charge) penetrate the mucus layer through the PEG layer, targeting F4/80^+^ macrophage populations via anti-F4/80 antibody, to deliver loaded siRNA into cytosol via F4/80^+^ receptor-mediated endocytosis following proton sponge effects of PEI. This process leads to macrophage-specific gene silencing with dramatic therapeutic efficacy in a DSS-induced murine colitis model. Another study prepared an orally delivered chitosan/alginate hydrogel that releases CD98 siRNA/PEI polyplex-loaded single-chain CD98 (scCD98) antibody functionalized chitosan nanoparticles with a positive net charge [77]. scCD98 antibody was used to target macrophages as well as intestinal epithelium, and the roles of the other components are the same as those in the above work. This system also showed good gene silencing activity in macrophages and intestinal epithelial cells both in vitro and in vivo, with dramatic therapeutic efficacy in a CD4^+^CD45RB^high^ T-cell transfer chronic colitis model. As another example, a degradable chitosan/alginate hydrogel loaded with IL-22 (pro-healing cytokine for the restoration of a disrupted intestinal barrier) and galactose (macrophage targeting moiety)-functionalized PLGA nanoparticles carrying anti-TNF-α with a net negative charge also showed good therapeutic efficacy in a DSS-induced mouse model of UC [104] (Figure 6). Three studies showed that a positive net charge is not necessary for active targeting functionalized nanoparticles. However, for efficient endosomal escape, an additional strategy such as the usage of PEI to perform the endosomal escape of complexed siRNA drugs via a proton sponge effect was utilized.

**Figure 7:**
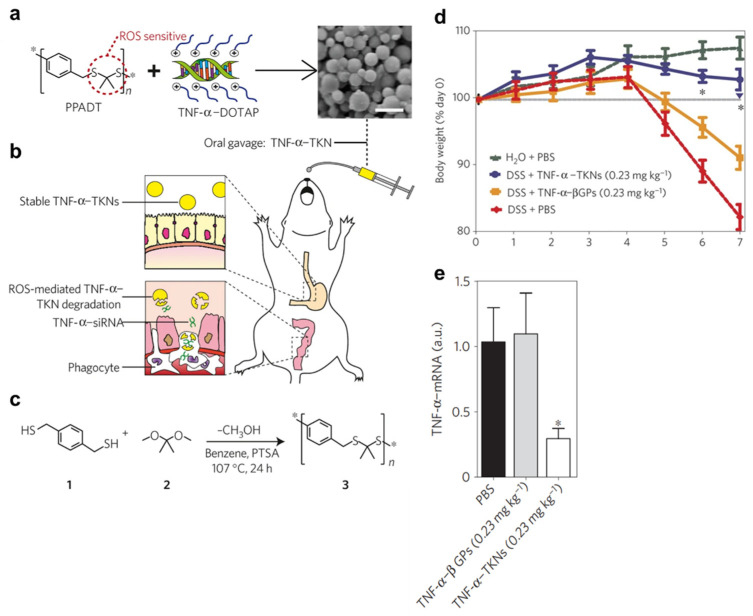
Polyplex nanoparticles embedded in ROS-responsive materials. (**a**): Poly-(1,4-pheyleneacetone dimethylene thioketal)(PPADT 3) is a new polymer composed of ROS-sensitive thioketal linkages (circled in red). The scanning electron micrograph shows TNF-α-loaded thioketal nanoparticles (TNF-α–TKNs) (scale bar represents 1.5 μm). (**b**): TNF-α–TKNs remain stable in the harsh environment of the gastrointestinal tract, while they degrade at the inflamed site where infiltrating phagocytes produce unusually high levels of ROS. Thus, TNF-α–siRNA is released at the inflamed site. (**c**): PPADT 3 was synthesized using the acetal exchange reaction. (**d**): Time course of mouse body weight. (**e**): Expression level of TNF-α mRNA decreased in the TNF-α–TKN treatment group. Statistical *p*-values: * p < 0.05. Reproduced with permission [108]. 2010, Springer Nature.

Inflamed site-specific release of the lipoplex or polyplex could be utilized to improve delivery and therapeutic efficacy [4]. The abnormally high levels of ROS produced at sites of intestinal inflammation could be exploited for inflamed site-specific release and action of polyplex or lipoplex [105,106,107]. Thioketal nanoparticles (TKNs) were developed by formulation from a polymer, poly-(1,4-pheyleneacetone dimethylene thioketal), which degrades selectively in response to ROS, encapsulating a polyplex composed of TNF-α siRNA complexed with DOTAP (1,2-dioleoyl-3-trimethylammonium-propane) [108] (Figure 7). The orally administered TKNs specifically release the polyplex at the inflamed site with higher levels of ROS, and the released polyplex specifically exerts strong siRNA gene silencing activity. Finally, these nanoparticles showed dramatic therapeutic efficacy in a DSS-induced murine colitis model, but the encapsulation of the polyplex in non-ROS-responsive nanoparticles instead of thio-ketal nanoparticles compromised the therapeutic efficacy, which is indicative of the crucial role of the ROS-mediated inflamed site-specific release of the polyplex in the therapeutic efficacy of the system.

The loading of the poly/lipoplex into additional materials could improve the stability of the system in the harsh GIT tract, but complexity issues hampering successful clinical translation should be considered.

## 4. Perspectives, Challenges, and Future Research Directions

siRNA-based drugs have shown great potential for both druggable and undruggable targets that could not be modulated using small molecule drugs or monoclonal antibodies. A plethora of research by academic researchers and pharmaceutical companies for the treatment of new or various diseases using siRNA has been performed over the last two decades. FDA-approved and commercially available siRNA-based drugs such as patisiran (ONPATTRO, a lipid nanoparticle-based siRNA drug for the treatment of polyneuropathy caused by hereditary transthyretin-mediated amyloidosis (hATTR)) [109,110], givosiran (GIVLAARI, a GalNAc-conjugated siRNA drug for the treatment of acute hepatic- or acute intermittent porphyria) [111], and lumasiran (OXLOMO, a GalNAc-conjugated siRNA drug for the treatment of primary hyperoxaluria type 1-kidney diseases) [112] are good models of siRNA delivery for the treatment of liver- or kidney-associated diseases. However, these drugs are based on parenteral routes. Compared with other routes of drug administration, oral administration is one of the most common due to the high patient compliance, simple self-administration, non-invasiveness, and low cost, but harsh GIT enzymes, mucus/intestinal epithelial barriers, and the penetration of lipid membranes of a cell are considerable challenges for siRNA drug delivery. Therefore, there has been extensive research for the development of various oral nanomedicines to protect siRNA drugs and specifically deliver them to the inflamed site and the intracellular cytosolic region. Unlike siRNA therapeutics administered by parenteral routes, orally administered siRNA-carrying nanomedicines have failed to reach the final market stage.

Despite progress over the last decade in oral nanomedicines, many challenges remain to be addressed for successful clinical translation. (1) First, siRNA intracytosolic delivery efficiency should be improved. To exert therapeutic efficacy, siRNA drugs should escape endosomes by some mechanism such as proton sponge effects and osmotic lysis. The efficiency of the endosomal escape of most currently developed nanomedicines is low, and, thus, further work is required to significantly improve efficiency. For example, current lipoid nanoparticles (LNPs), which are the most successful vehicles for RNA (mRNA, siRNA) drug delivery in the clinic, generally have a low degree of endosomal escape (< 2%) [83]. A pH-responsive lipid or polymer-based lipoplex or polyplex could be utilized for efficient intra-cytosol delivery [83]. (2) In addition, complexity issues should be addressed. Many oral nanomedicines carrying siRNA drugs have complex structures that make it difficult to overcome the various barriers mentioned above. Thus, difficulty in the large-scale preparation of nanoparticles and associated quality control issues could impede clinical translation. Furthermore, a multitude of components leads to an increase in production cost, which should be considered. (3) Even though siRNA-mediated therapeutics are effective in the beneficial management of disrupted intestinal barriers and dysregulated immune systems in IBD treatment, the beneficial manipulation of the dysbiotic gut microbiome should be considered [4,5,113,114,115], and this is hard to address with siRNA drugs. The development of oral nanomedicines targeting all these factors simultaneously may lead to improved results in IBD management, since considerable recent research has shown that all three factors are crucial in the induction, severity, and progression of inflammatory bowel diseases. For example, we recently demonstrated an oral nanomedicine, hyaluronic acid-bilirubin nanomedicine (HABN), which is capable of targeting and beneficially modulating disrupted intestinal barriers, dysbiotic gut microbiota, and dysregulated immune systems while exerting potent anti-inflammatory efficacy in a murine colitis model of DSS [5]. Orally administered HABN significantly normalized the diversity of the gut microbiome and increased the relative abundance of *Akkermansia muciniphila* (known to induce a protective intestinal barrier), *Clostridium XIV* *α* (known to induce regulatory CD4 T-cells), and *Lactobacillus* (known to have anti-inflammation effects). Notably, the therapeutic efficacy of HABN was significantly abrogated by the antibiotic-pretreated depletion of gut microbiota, which is indicative of its crucial role in HABN-based therapy. Therefore, when designing siRNA-based nanomedicines, gut microbiome modulation should be considered. (4) The safety issues of nanomaterials should also be addressed. Since IBD requires long-term therapeutic intervention, the safety profiles of nanomaterials carrying siRNA drugs should be thoroughly investigated. (5) Over recent decades, it was revealed that the plasma protein corona on the surface of nanomedicines administered intravenously has a crucial role in the targeting ability, distribution, and mode of action (MOA) of nanoparticles. Likewise, orally administered nanomedicines may form a protein corona with various intestinal proteins derived from host, foods, and other environments, which also affects the targeting ability, distribution, and activity of the nanoparticles.

In conclusion, despite the many challenges in clinically translating siRNA therapeutics, the approval of three siRNA-based targeted therapeutics within four years could accelerate the development and approval of new classes of siRNA therapeutics. Among the new generation of such drugs, oral nanomedicines carrying siRNA are a very attractive option to treat diseases, especially IBD and colorectal cancer (CRC), due to the advantages of oral administration compared to parenteral routes. Therefore, effort will be expended into additional research leading to the design, development, and clinical testing of additional oral nanomedicines carrying siRNA drugs. After addressing the challenges indicated above, these systems will become useful tools for the treatment of various intestinal diseases such as IBD and CRC.

## Figures and Tables

**Table 1:** Strategies for the oral delivery of siRNA drugs.

siRNA Delivery Methods		Advantages	Drawbacks
Chemical modification		- Improve stability against enzyme - Increase resistance of siRNA to enzymatic attack - Sufficient to protect small sized siRNA drugs against systemic metabolism, leading to enhancement of drug activity	- Cannot garantee enhanced intracellular delivery of siRNA - Some siRNA drugs (e.g., larger siRNA drugs) are not sufficiently stabilized
Co-administration of nuclease inhibitors		- Improve stability - Immobilized onto polymers of the drug carrier matrix: localize the inhibitory activity	- Cannot guarantee enhance intracellular delivery of siRNA
Lipo-polyplex formation	-	- Protect siRNA against harsh conditions of GIT - Enhance targeting ability - Improve half-life - Can apply to siRNA drugs that are not sufficiently stabilized by chemical modification and larger siRNA drugs - Leads to obstruction of free access of nucleases to nucleic acid-based therapeutics due to increased steric hindrance via nanoparticle formation - Enhanced intracellular uptake due to increased lipophilicity - Easily penetrate into the remaining mucus layer of the inflamed tissue - No need to be smaller than 100 nm and muco-inert to penetrate the mucus gel layer	- Escape from endo/lysosomal vesicles is the limiting step - Can be trapped in the mucus layer - Could be non-specifically internalized into many kinds of cells after penetration of the mucus layers - Non-specifically bound to healthy intestinal barrier due to charge-charge interactions - PEG coating: might hinder intracellular uptake of nanomedicines - Strong negative net charge: hinder intracellular uptake due to charge-charge repulsion - Neutral net surface charge: cannot escape from endo/lysosomal vesicles -> degradation of siRNA
	With targeting ligands	- Further improve drug targeting to the sites - Can be taken up by specific cells regardless of their net charge. - Could induce target cell-specific distribution and enhance cellular membrane penetration via receptor-mediated endocytosis.	- Possibility for the lipoplex or polyplex to non-specifically bind to healthy intestinal barrier, especially the mucus layer.
Lipo-polyplex embedded in hydrogels or polymeric materials	-	- Further impede free access of nucleases to siRNA drugs: further protect against nuclease attack - Increases the stability of loaded siRNA drugs in the harsh environment by providing an additional protective layer	- Robust loading of the polyplex or lipoplex could hamper efficient release of loaded materials from the embedding materials.
	With targeting ligands	- Improve target cell-specific distribution after release of the nanomaterials from the embedding material. - Enhanced cellular membrane permeation via receptor-mediated endocytosis.	- For efficient endosomal escape, an additional strategy such as usage of PEI to perform endosomal escape of complexed siRNA drugs should be utilized - Complexity issues hampering successful clinical translation

## Data Availability

Not applicable.

## Abbreviations

ANP	Aminated nanoparticle
CD	Crohn’s disease
CNP	Carboxylated nanoparticle
CRC	Colorectal cancer
DEAEMA	Diethylaminoethyl Methacrylate
DOPA	1,2-dioleoyl-sn-glycero-3-phosphate
DOTA	1,4,7,10-tetraazacyclododecane1,4,7,10-tetraacetic acid
DOTAP	1,2-dioleoyl-3-trimethylammonium-propane
DSPC	Distearoylphosphatidylcholine
DSS	Dextran sodium sulfate
EDTA	Ethylenediamine tetraacetic acid
GAPDH	Glyceraldehyde-3-Phosphate Dehydrogenase
GDLN	Ginger-derived lipid nanoparticle
GeRP	Glucan-Encapsulated siRNA Particle
GI	Gastrointestinal
GIT	Gastrointestinal tract
HABN	Hyaluronic acid-bilirubin nanomedicine
hATTR	Hereditary transthyretin-mediated amyloidosis
IBD	Inflammatory bowel disease
ICAM-1	Intercellular adhesion molecule-1
LNP	Lipoid nanoparticles
LPS	Lipopolysaccharide
Ly6C	Lymphocyte antigen 6 complex
MAA	Methacrylic acid
MOA	Mode of action
MPO	Myeloperoxidase
NiMOS	Nanoparticles-in-microsphere oral system
NVP	N-Vinylpyrrolidone
PCL	poly(epsilon-caprolactone)
pDNA	Plasmid DNA
PEC	Polyelectrolyte complex
PEG	Polyethylene glycol
PEI	Polyethyleneimine
PLGA	Poly(lactic-co-glycolic acid)
PNP	Plain nanoparticle
RNAi	RNA interference
ROS	Reactive oxygen species
scCD98	Single-chain CD98
SEDDS	Self-emulsifying drug delivery system
siRNA	Small interfering RNA
TKN	Thioketal nanoparticle
UC	Ulcerative colitis
